# Low Hartmann’s procedure versus abdominoperineal resection for rectal cancer, a propensity score matching cohort study

**DOI:** 10.1186/s12876-024-03244-5

**Published:** 2024-06-05

**Authors:** Xubing Zhang, Shaojun Liu, Liu Liu, Zhiqiang Zhu

**Affiliations:** Department of Gastrointestinal Surgery, the First Affiliated Hospital of USTC Division of Life Sciences and Medicine, University of Science and Technology of China, Lujiang Road No. 17, Hefei, 230001 China

**Keywords:** Rectal cancer, Low hartmann’s procedure, Abdominoperineal resection, Postoperative complication, Pelvic abscess

## Abstract

**Background:**

This study aimed to compare low Hartmann’s procedure (LHP) with abdominoperineal resection (APR) for rectal cancer (RC) regarding postoperative complications.

**Method:**

RC patients receiving radical LHP or APR from 2015 to 2019 in our center were retrospectively enrolled. Patients’ demographic and surgical information was collected and analyzed. Propensity score matching (PSM) was used to balance the baseline information. The primary outcome was the incidence of major complications. All the statistical analysis was performed by SPSS 22.0 and R.

**Results:**

342 individuals were primarily included and 134 remained after PSM with a 1:2 ratio (50 in LHP and 84 in APR). Patients in the LHP group were associated with higher tumor height (*P* < 0.001). No significant difference was observed between the two groups for the incidence of major complications (6.0% vs. 1.2%, *P* = 0.290), and severe pelvic abscess (2% vs. 0%, *P* = 0.373). However, the occurrence rate of minor complications was significantly higher in the LHP group (52% vs. 21.4%, *P* < 0.001), and the difference mainly lay in abdominal wound infection (10% vs. 0%, *P* = 0.006) and bowel obstruction (16% vs. 4.8%, *P* = 0.028). LHP was not the independent risk factor of pelvic abscess in the multivariate analysis.

**Conclusion:**

Our data demonstrated a comparable incidence of major complications between LHP and APR. LHP was still a reliable alternative in selected RC patients when primary anastomosis was not recommended.

## Introduction

Rectal cancer (RC) ranks top three malignancies worldwide, threatening to patients’ quality of life and survival [1]. In the past decades, significant progress has been achieved in multimodality therapy for RC. However, radical surgery, with the criterion of total mesorectal excision (TME), still plays a crucial role in the combined modality treatment for RC [2]. Several surgical procedures have been applied for RC, including anterior resection (AR) and intersphincteric resection (ISR) with sphincter preserving, while abdominoperineal resection (APR) with permanent end colostomy [3].

Besides the procedures described above, “Hartmann’s procedure” is another surgical approach for RC. Professor Hartmann firstly proposed it in 1923 [4]. Due to distal rectum closure and proximal endo-colostomy, it was traditionally regarded as one safer procedure and was often performed for those with a poor physical condition. It was associated with less surgical trauma and faster recovery [5]. In recent years, the term “low Hartmann’s procedure (LHP)” has been proposed, and it mainly refers to the procedure for tumors located within 10 cm of the anal verge [6].

APR was once adopted as the standard procedure for low-lying RC, but it was associated with a higher incidence of postoperative complications, especially perineal wound infection [7]. APR has been significantly less performed in recent years, with the advances of surgical technique and the emphasis on organ preservation [8]. However, low or even ultra-low anastomosis is associated with an increased risk of anastomotic leakage [9]. Sometimes, primary anastomosis might not be the best choice, especially for those with poor blood supply to the residual bowel or with poor physical condition. As a result, LHP was proposed as an alternative in those situations [10].

Until now, literature directly comparing LHP with APR is still limited [11]. Besides, the surgical-related outcomes varied among studies. Some indicated that LHP was associated with a higher incidence of postoperative pelvic abscess, and a higher frequency of reoperation and readmission when compared with APR [12]. However, these results were not supported by some other literature. Thus, we conducted this study to compare LHP with APR regarding postoperative complicationswith the data in our prospective database. We hope our study can provide more reference to current practice.

## Materials and methods

### Patients

This study was retrospective and case-control designed, with the patients in the Department of Gastrointestinal Surgery, the First Affiliated Hospital of USTC (Anhui Provincial Hospital), from January 2015 to December 2019. Patients with pathologically confirmed RC and receiving LHP or APR were enrolled. The exclusion criteria were listed as follows: (1) tumors of which the lower edge is located beyond 10 cm to the anal verge; (2) other kinds of rectal tumors including stromal tumor, neuroendocrine neoplasm, and malignant melanoma; (3) patients with a history of malignant tumors in the gastrointestinal tract and pelvic; (4) emergency surgery; This study was approved by the Ethics Committee of the First Affiliated Hospital of USTC. Informed consent was obtained from all the patients.

### Data collection

All the data were stored and updated in the prospective database in our center. The following data were collected:

Baseline information: age, gender, body mass index (BMI), previous abdominal surgery (PAS), the distance between the lower edge of the tumor and anal verge, neoadjuvant chemoradiotherapy (neoCRT), ASA score, and pathological outcomes.

Surgical information: surgical procedure, operative time, estimated blood loss, intraoperative blood transfusion, and the detail of combined organ resection.

Postoperative recovery: time to first flatus, time to first fluid diet, postoperative hospital stays, postoperative blood transfusion, and ICU stay.

Postoperative complications were divided into a short-term group (within 90 days) and a long-term group (beyond 90 days) according to previous studiesAll the complications were evaluated based Clavien-Dindo Classification and classified as major and minor complication. The incidence of reoperation (within 90 days), readmission (within 90 days) and mortality (within 90 days) were also collected. The diagnosis of pelvic infection must have the support of the etiology. The severe pelvic abscess was defined as these needing CT or ultrasound-guided puncture and drainage or for which re-operation was performed.

### Surgical procedure

Senior colorectal surgeons in our center performed all the surgery with more than ten years’ experience. The choice of LHP or APR was mainly based on the patient’s physical condition, risk of anastomotic complications, and anal function. Besides, surgeon’s experience also provided a reference. The laparoscopic approach was the first choice. When the surgery is finished, catheterization with double lumen would be placed in the pelvis for drainage, extracted through the abdominal wall in LHP and trans-perineal in APR.

### Statistical analysis

Continuous variables were expressed as median (range) or mean (standard deviation), and a non-parametric Mann–Whitney U test or independent-sample t-test was induced for analysis. Ranked data were also analyzed by non-parametric test. Categorical variables were shown as a number and analyzed by Chi-Square or Fisher’s exact tests. Logistic regression was used to analyze the factors related to postoperative complications. According to some previous studies, propensity score matching (PSM) was performed by R (version 4.4.4), with a 1:2 ratio and Caliper = 0.02, based on gender, age, tumor stage, ASA score, BMI, PAS, and surgical procedure. The percentage of patients with neoCRT was extremely low and no significant difference was found between the two groups, so we did not include neoCRT for PSM. A p-value < 0.05 was deemed to be significant. All statistical analysis was performed using SPSS 22.0.

## Results

### Patients’ characteristic

**Fig. 1:**
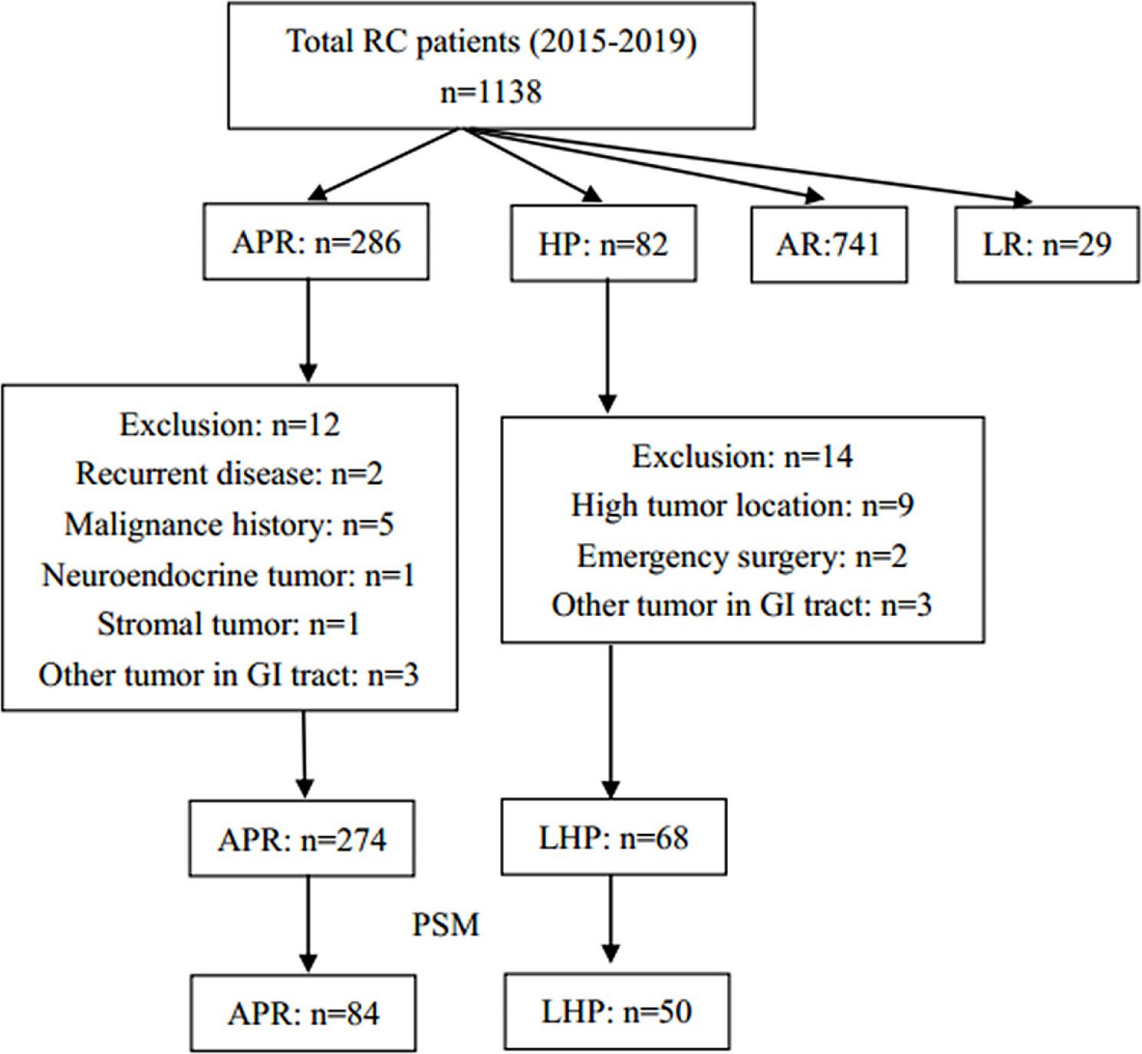
Patients selection flow. RC, rectal cancer; APR, abdominoperineal resection; LHP, low Hartmann’s procedure; AR, anterior resection; LR, local resection; GI, gastrointestinal

**Fig. 2:**
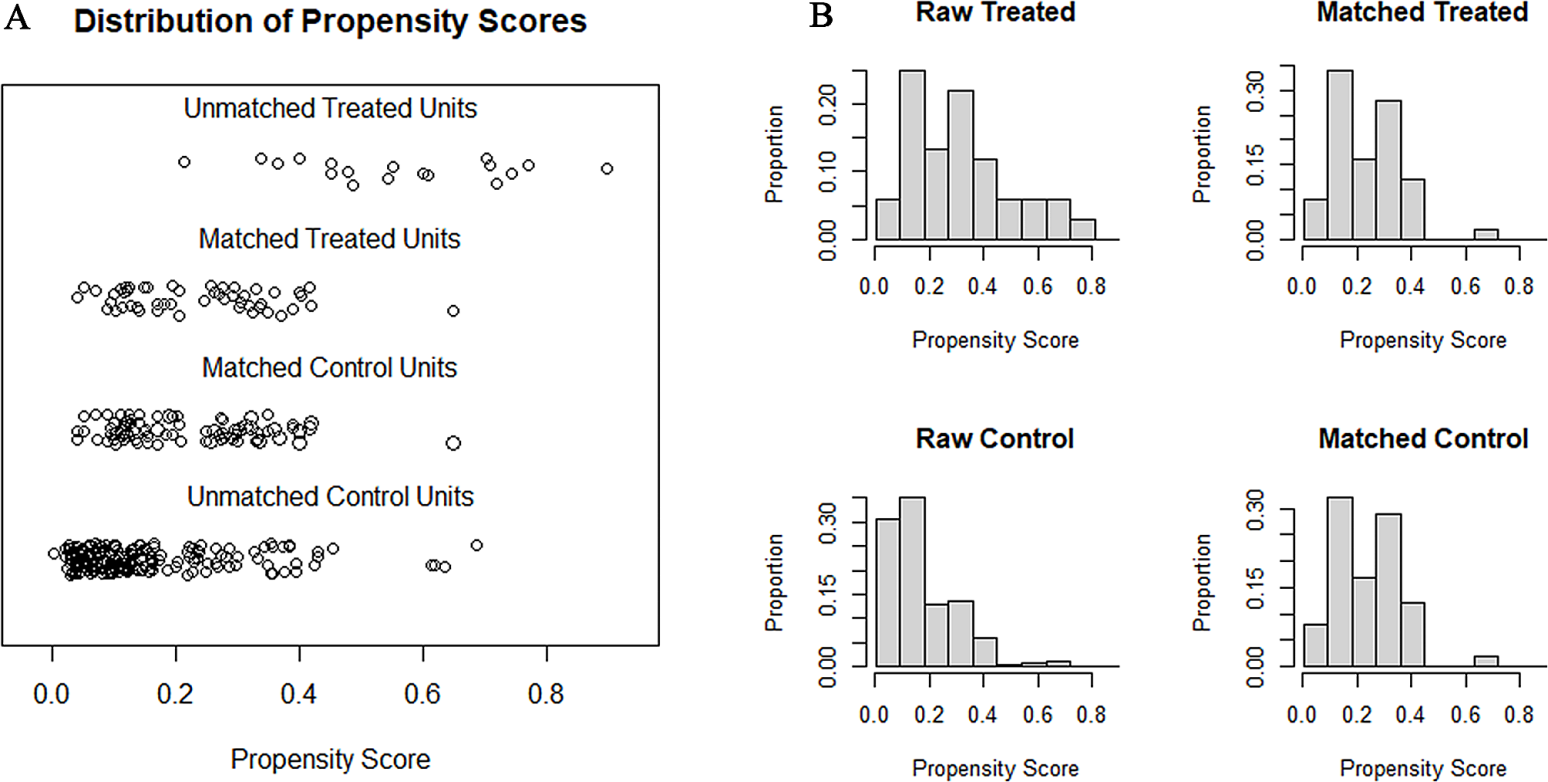
The effect picture of propensity score matching (PSM) (**A**, jitter plot; **B**, hist)

The patient selection process was shown in Fig. 1, and the demographic information was shown in Table 1. Before matching, 342 patients were primarily enrolled in this study, and among them, 68 patients received LHP and the other 274 patients received APR. Patients in the LHP group showed significantly older age (median 66.5 vs. 64, *P* = 0.002), higher tumor location (median 6.0 vs. 3.0, *P* < 0.001), higher ASA score (*P* = 0.011), and advanced tumor stage (*P* = 0.009). After PSM with a 1:2 ratio, 134 patients remained (50 in the LHP group and 84 in the APR group). Except for tumor location (*P* < 0.001), other baseline information was comparable between the two groups. The jitter plot and hist showed an appropriate effect of PSM. (Fig. 2)

**Table 1:** Patients’ demographics

	Before matching			After matching		
variable	LHP (*n* = 68)	APR (*n* = 274)	*P* value	LHP (*n* = 50)	APR (*n* = 84)	*P* value
Gender			0.381			0.777
male	38 (55.9%)	169 (61.7%)		31	50	
female	30 (44.1%)	105 (38.3%)		19	34	
Age (year)	66.5 (28–92) *	64.0 (22–89) *	**0.002**	66 (28–89) *	66 (31–89) *	0.653
	67.5 ± 12.9 **	62.1 ± 12.3 **		65.0 ± 12.2 **	65.4 ± 11.1 **	
BMI (Kg/m^2^)	22.7 (16.4–30.8) *	23.2 (14.7–33.3) *	0.356	23.44 (17.58–30.82) *	23.76 (14.67–33.33) *	0.565
	22.9 ± 3.2 **	23.3 ± 3.2 **		23.3 ± 3.2 **	23.6 ± 3.6 **	
PAS	14 (20.6%)	34 (12.4%)	0.082	7 (14%)	11 (13.1%)	0.882
Distance to anal verge (cm)	6.0 (2–10)	3.0 (1–7)	**< 0.001**	7 (2–10)	3 (1–6)	*P* < 0.001
neoCRT	5 (7.4%)	14 (5.1%)	0.470	2 (4%)	1 (1.2%)	0.646
ASA score			**0.011**			0.707
1	0	4 (1.5%)		0	1 (1.2%)	
2	20 (29.4%)	131 (47.8%)		20 (40%)	31 (36.9%)	
3	48 (70.6%)	139 (50.7%)		30 (30%)	52 (61.9%)	
Pathological outcomes			**0.009**			0.306
0	0	7 (%)		0	0	
I	6 (8.8%)	74 (%)		6 (12%)	16 (19.0%)	
II	22 (32.4%)	79 (%)		17 (34%)	21 (25%)	
III	39 (57.4%)	113 (40.1%)		26 (52%)	47 (56.0%)	
IV	1 (1.5%)	1 (0.4%)		1 (2%)	0	

LHP, low hartmann’s procedure; APR, abdominoperineal resection; BMI, body mass index; PAS, previous abdominal surgery; neoCRT, neoadjuvant chemoradiotherapy; ASA, American Society of Anesthesiologists

*median (min = max); ** mean ± SD

### Intraoperative and postoperative outcomes

Surgical details are provided in Table 2. After PSM, no significant difference was observed between the two groups regarding the application of laparoscopic technique (86% vs. 89.3%, *P* = 0.571), operative duration (185 min vs. 200 min, *P* = 0.314), or estimated blood loss (100 ml vs. 100 ml, *P* = 0.191). Besides, a comparable occurrence rate of intraoperative blood transfusion (2% vs. 0, *P* = 0.373) and combined organ resection (8% vs. 2.4%, *P* = 02.76) was also observed between the two groups.

**Table 2:** Operative information

	Before matching			After matching		
Variables	LHP (*n* = 68)	APR (*n* = 274)	*P* value	LHP (*n* = 50)	APR (*n* = 84)	P value
Surgical procedure			**< 0.001**			0.571
laparoscopy	52 (76.5%)	251 (91.6%)		43 (86%)	75 (89.3%)	
open	16 (23.5%)	23 (8.4%)		7 (14%)	9 (10.7%)	
Operative time (min)	184 (80–420)	200 (95–420)	**0.012**	185 (80–420)	200 (110–365)	0.314
Blood loss (ml)	100 (10–400)	100 (20–800)	0.340	100 (10–400)	100 (20–400)	0.191
Intraoperative blood transfusion	1 (1.5%)	2 (0.7%)	1.000	1 (2%)	0	-
Combined resection	5 (7.4%)	3 (1.1%)	**0.009**	4 (8%)	2 (2.4%)	0.276
Liver	1	0		1	0	
Cholecyst	1	0		1	0	
Uterus	1	0		1	0	
Vagina	1	0		1	0	
Ovary	1	1		1	0	
Bowel	0	1		0	1	
Bladder	0	1		0	1	
Time to first flatus (day)	2.0 (0.5-5.0)	2.0 (0.5-6.0)	0.794	2.0 (0.5-4.0)	2.0 (0.5-5.0)	0.698
Time to first diet (day)	3.0 (1.5-8.0)	4.0 (1.0–9.0)	0.266	3.0 (1.5-6.0)	4.0 (1.5-7.0)	0.440
Postoperative hospital stay (day)	8 (4–26)	8 (4–33)	0.969	8 (4–26)	9 (4–19)	0.514
Postoperative blood transfusion	9 (13.2%)	11 (4.0%)	**0.004**	6 (12%)	3 (3.6%)	0.126
Postoperative complications (short-term)	32(47.1%)	73(26.6%)	**0.001**	26 (52%)	18 (21.4%)	**< 0.001**
Major (Clavien-Dindo III - IV)	4 (5.9%)	8 (2.9%)	0.235	3 (6%)	1 (1.2%)	0.290
Bowel obstruction	1	4	1.000	1	1	1.000
Stoma related	1	1	0.359	1	0	0.373
Pelvic abscess	2	1	0.102	1	0	0.373
Cerebral infarction	1	1	0.359	0	0	-
Wound bleeding (perineal)	-	1	-	-	0	-
Minor (Clavien-Dindo I- II)	32 (47.1%)	66 (24.1%)	**< 0.001**	26 (52%)	18 (21.4%)	**< 0.001**
Pelvic infection	8	32	0.984	8	9	0.374
Pulmonary infection	6	6	**0.008**	5	2	0.130
Wound infection (abdominal)	7	5	**0.001**	5	0	**0.006**
Wound infection (perineal)	-	21	-	-	4	-
Pelvic bleeding	0	1	-	0	0	-
Bowel obstruction	10	5	**< 0.001**	8	4	**0.028**
Chylous leakage	3	1	**0.026**	3	0	**0.050**
Urinary infection	1	3	1.000	1	1	1.000
Urinary dysfunction	5	3	**0.009**	3	0	**0.050**
Thrombosis	1	0	-	1	0	0.373
Stoma-related	0	1	-	0	0	-
ICU stay	7	5	**0.001**	3	3	0.822
Reoperation (within 90 days)	2	6	1.000	2	1	0.646
Readmission (within 90 days)	3	6	0.548	3	0	**0.050**
Mortality (within 90 days)	0	0	-	0	0	-
Postoperaive complication (long-term)	4 (5.9%)	18 (6.6%)	1.000	2 (4%)	8 (9.5%)	0.403
Major (Clavien-Dindo III - IV)	4 (5.9%)	16 (5.8%)	1.000	2 (4%)	7 (9.5%)	0.540
Parastomal hernia	2	8	1.000	1	6	0.372
Perineal hernia	-	3	-	-	0	-
Stoma prolapse	1	0	-	0	0	-
Bowel obstruction	1	2	0.478	1	1	
Wound infection (perineal)	-	1	-	-	0	-
Incision dehiscence (perineal)	-	1	-	-	0	-
Minor (Clavien-Dindo I- II)	0	2(0.7%)	-	0	1 (1.2%)	-
Parastomal hernia	0	1	-	0	0	-
Bowel obstruction	0	1	-	0	1	-

LHP, low hartmann’s procedure; APR, abdominoperineal resection

When it came to postoperative recovery, no significant difference was observed between the two groups in terms of time to first flatus (2.0d vs. 2.0d, *P* = 0.0.698), time to first fluid diet (3.0d vs. 4.0d, *P* = 0.440), and postoperative hospital stays (8d vs. 9d, *P* = 0.514). (Table 2)

A total of 26 (52%) and 18 (21.4%) patients developed short-term postoperative complications in the LHP and APR groups, respectively (*P* < 0.001). Although the incidence of major complications was higher in the LHP group, that was not significantly different (6% vs. 1.2%, *P* = 0.290). The most common major complication in the whole cohort was bowel obstruction (1 in the LHP group (2%), 1 in the APR group (1.2%), *P* = 1.000). Besides, a severe pelvic abscess was observed in one individual (2%) in the LHP group, and it was well managed by ultrasound-guided percutaneous peritoneal drainage. Nevertheless, the total incidence of minor complications was significantly higher in the LHP group than that in the APR group (52% vs. 24.1%, *P* < 0.001). The disparity was mainly reflected in abdominal wound infection (10% vs. 0, *P* = 0.001) and bowel obstruction (16% vs. 4.8%, *P* < 0.028). The most common minor complication was pelvic infection, and the incidence was comparable between the two groups (16% vs. 10.7%, *P* = 0.374). All the pelvic infection was well managed by antibiotics and no further intervention was needed. Besides, similar outcomes were observed between the two groups for the incidence of ICU stay (6% vs. 3.6%, *P* = 0.822), reoperation (4% vs. 1.2%, *P* = 0.646), and readmission (6% vs. 0, *P* = 0.05). No perioperative mortality occurred in both groups. (Table 2)

The median follow-up time for patients developing long-term complications were 37 (25–63) months. No significant difference was observed between the two groups for the incidence of long-term complications (4% vs. 9.5%, *P* = 0.403), either for the major or minor classification. As our observation, in this cohort, the most common long-term complication was parastomal hernia, especially in the APR group, and it was the main reason for readmission and reoperation. Besides, the earliest occurrence of parastomal hernia was ten months after surgery. (Table 2)

### Risk factor analysis for postoperative complications (short-term)

In the univariate analysis, we found that the tumor located within 5 cm of the anal verge (*P* = 0.002, OR = 2.154, 95%CI [1.312, 3.535]) and LHP (*P* = 0.001, OR = 2.494, 95%CI [1.443,4.309]) were significantly associated with increased incidence of short-term complications. As tumor location was significantly associated with the surgical procedures, we excluded tumor location from the multivariate analysis. The multivariate analysis demonstrated that increased blood loss (*P* = 0.048, OR = 1.796, 95%CI [1.005, 3.208]) and LHP (*P* = 0.001, OR = 4.246, 95% CI [1.750, 10.302]) were independent risk factors for postoperative complications. (Table 3)

**Table 3:** Univariate and multivariate analysis for postoperative complications (short-term) with PSM data

Variables	Univariate analysis			Multivariate analysis		
	OR	95%CI	P value	OR	95%CI	P value
Gender (female/male)	1.413	0.667, 2.993	0.367	1.838	0.711, 4.751	0.209
Age (< 65/≥65y)	1.215	0.582, 2.536	0.605	1.261	0.481, 3.307	0.637
BMI (< 24/≥24Kg/m2)	1.308	0.634, 2.695	0.467	1.181	0.460, 3.037	0.729
PAS (no/yes)	1.778	0.648, 4.879	0.264	2.093	0.556, 7.875	0.275
ASA score (1,2/3)	1.165	0.540, 2.511	0.697	1.165	0.395, 3.437	0.782
Distance to anal verge(≥ 5/<5 cm)	0.270	0.127, 0.575	**0.001**	-	-	-
Surgical approach (open/laparoscopy)	0.792	0.268, 2.339	0.673	1.896	0.367, 9.787	0.445
Surgical procedure (APR/LHP)	3.972	1.855, 8.504	**< 0.001**	4.246	1.750, 10.302	**0.001**
Operative time (< 195/≥195 min)	0.853	0.412, 1.767	0.669	0.536	0.189, 1.520	0.241
Blood loss (< 100/≥100 min)	1.427	0.655, 3.108	0.371	2.888	1.043, 7.995	**0.041**
Combined organ resection (no/yes)	2.122	0.410,10.970	0.369	1.920	0.231, 15.978	0.546
ICU stay (no/yes)	2.122	0.410,10.970	0.369	1.140	0.130, 10.035	0.906
Perioperative blood transfusion (no/yes)	3.395	0.905, 12.727	0.070	2.250	0.475, 10.671	0.307
Pathological stage (I,II/III,IV)	1.047	0.505, 2.173	0.901	1.377	0.570, 3.329	0.477

LHP, low hartmann’s procedure; APR, abdominoperineal resection; BMI, body mass index; PAS, previous abdominal surgery; neoCRT, neoadjuvant chemoradiotherapy; ASA, American Society of Anesthesiologists; PSM, propensity score matching

We further explored the risk factors associated with postoperative pelvic abscess/infection. Unfortunately, no independent risk factor was found in univariate or multivariate analysis. (Table 4)

**Table 4:** Univariate and multivariate analysis for pelvic abscess/infection with PSM data

Variables	Univariate analysis			Multivariate analysis		
	OR	95%CI	P value	OR	95%CI	P value
Gender (female/male)	1.143	0.419, 3.119	0.794	1.688	0.518, 5.505	0.385
Age (< 65/≥65y)	0.600	0.226, 1.590	0.304	0.693	0.219, 2.194	0.533
BMI (< 24/≥24Kg/m2)	0.659	0.242, 1.794	0.414	0.701	0.219, 2.246	0.550
PAS (no/yes)	1.250	0.325, 4.809	0.745	1.098	0.223, 5.413	0.908
ASA score (1,2/3)	0.821	0.305, 2.212	0.696	0.805	0.215, 3.022	0.748
Distance to anal verge(< 5/≥5 cm)	0.714	0.269, 1.894	0.499	-	-	-
Surgical approach (open/laparoscopy)	0.680	0.174, 2.652	0.578	2.041	0.302, 13.793	0.464
Surgical procedure (LHP/APR)	0.791	0.295, 2.122	0.642	1.036	0.350, 3.064	0.949
Operative time (< 195/≥195 min)	0.642	0.240, 1.717	0.378	0.380	0.103, 1.400	0.146
Blood loss (< 100/≥100 min)	1.656	0.557, 4.927	0.346	2.055	0.575, 7.343	0.268
Combined organ resection (no/yes)	3.265	0.555, 19.214	0.191	6.584	0.623, 69.565	0.117
Perioperative blood transfusion (no/yes)	1.574	0.308, 8.046	0.586	1.201	0.182, 7.927	0.849
Pathological stage (I,II/III,IV)	1.091	0.408, 2.918	0.862	1.140	0.392, 3.316	0.810

LHP, low hartmann’s procedure; APR, abdominoperineal resection; BMI, body mass index; PAS, previous abdominal surgery; neoCRT, neoadjuvant chemoradiotherapy; ASA, American Society of Anesthesiologists; PSM, propensity score matching

Then we continued to explore the risk factors associated with bowel obstruction. In the univariate analysis, higher tumor location (*P* = 0.034, OR = 3.800, 95%CI [1.106, 13.055]), LHP (*P* = 0.019, OR = 4.390, 95%CI [1.275, 15.118]), and perioperative blood transfusion (*P* = 0.038, OR = 4.886, 95%CI [1.091, 21.875]) were significantly associated with higher incidence of bowel obstruction. In the multivariate analysis, LHP (*P* = 0.013, OR = 11.685, 95%CI [1.688, 80.876]) and increased blood loss (*P* = 0.028, OR = 12.922, 95%CI [1.318, 126.652]) were independent risk factors of bowel obstruction. Besides, prolonged operative time (*P* = 0.044, OR = 0.105, 95%CI [0.012, 0.942]) was one protective factor. (Table 5)

**Table 5:** Univariate and multivariate analysis for bowel obstruction with PSM data

Variables	Univariate analysis			Multivariate analysis		
	OR	95%CI	P value	OR	95%CI	P value
Gender (female/male)	2.347	0.615, 8.965	0.212	4.928	0.799, 30.397	0.086
Age (< 65/≥65y)	1.696	0.495, 5.810	0.401	2.299	0.384, 13.783	0.362
BMI (< 24/≥24Kg/m2)	0.939	0.794, 1.112	0.467	2.420	0.450, 13.021	0.303
PAS (no/yes)	2.120	0.523, 8.588	0.292	2.374	0.340, 16.601	0.383
ASA score (1,2/3)	1.257	0.357, 4.423	0.721	0.466	0.061, 3.581	0.463
Distance to anal verge(< 5/≥5 cm)	3.800	1.106, 13.055	**0.034**	-	-	-
Surgical approach (open/laparoscopy)	1.698	0.206, 14.012	0.623	9.846	0.414, 234.133	0.157
Surgical procedure (APR/LHP)	4.390	1.275, 15.118	**0.019**	11.685	1.688, 80.876	**0.013**
Operative time (< 195/≥195 min)	0.556	0.172, 1.797	0.326	0.105	0.012, 0.942	**0.044**
Blood loss (< 100/≥100 min)	1.273	0.370, 4.380	0.702	12.922	1.318, 126.652	**0.028**
Perioperative blood transfusion (no/yes)	4.886	1.091, 21.875	**0.038**	7.443	0.958, 57.809	0.055
Pathological stage (I,II/III,IV)	0.453	0.140, 1.467	0.186	0.371	0.078, 1.775	0.214

LHP, low hartmann’s procedure; APR, abdominoperineal resection; BMI, body mass index; PAS, previous abdominal surgery; neoCRT, neoadjuvant chemoradiotherapy; ASA, American Society of Anesthesiologists

## Discussion

LHP was once adopted as an alternative to APR for RC when primary anastomosis was not recommended [13]. However, it’s still inconclusive whether LHP is one good choice because a higher incidence of complications after LHP was observed in some literatures, especially the high occurrence rate of pelvic abscess [14]. Therefore, we conducted this study and reviewed the data in our center to compare LHP with APR in terms of surgical-related outcomes by PSM.

In previous reports, the occurrence rate of pelvic abscess after LHP ranged from 3.7 to 30% [15, 16]. However, none distinguished severe pelvic abscess from general one according to the Clavien-Dindo classification. Interestingly, an extremely low incidence of severe pelvic abscesses was observed in our cohort, which was comparable between the two groups. We thought the applying wide-spectrum antibiotics and pelvic drainage contributed to the low incidence [17]. Besides, the total rate of pelvic abscesses or infection (13.4%) in our study was comparable to that in previous literature [18]. According to previous reports, the occurrence rate of pelvic abscess after APR was also discrepant among studies [19]. For instance, Frye et al. reported no pelvic abscesses after APR, while that was 17.2% in the LHP group [15].

In contrast, Sverrisson et al. indicated a significantly higher incidence of pelvic-related complications after APR compared to that in the LHP group (32% vs. 13%, *P* < 0.001). This might be due perineal wound infection in the APR group was also counted as pelvic-related complications [12]. However, up to now, the reason for the high incidence of pelvic abscess after LHP in some studies was still not well indicated. It was proposed that the rectal stump after LHP was a potential risk for pelvic abscess [20]. Therefore, trans-anal wash before stapling might be essential since a reinforced suture for residual stump seems technically difficult. However, with the advancement of the stapling technique, the leakage of the rectal stump after LHP has been rarely reported in recent years.

As reported, the incidence of perineal wound infection after APR ranged from 15 to 47%, and it was associated with delayed healing and poor quality of life [21]. Besides, perineal wound infection might add to the risk of pelvic abscess. A relatively low rate of perineal wound infection (4.8%) after APR was observed in our study, compared with 21.4% in Rodríguez’s and 14.3% in Frye’s reports [15, 18]. This should be attributed to the advancement of perioperative care and trans-perineal pelvic drainage [22], as we also observed the decreasing incidence of perineal wound infection in recent literature. Although LHP can avoid perineal wound when compared with APR, it still needs an incision at the hypogastrium. Interestingly, about 10% incidence of abdominal wound infection was observed in the LHP group. It seems that LHP has no obvious advantages when regarding the incidence of wound infection. However, we still think perineal wound infection is harder to handle and had longer time to heal.

Although the incidence of major complications was comparable between the two groups, a significantly higher rate of minor complications was still observed in the LHP group. However, the potential reason for this outcome still cannot be well indicated. Most previous studies did not divide the complications into major and minor groups. One previous study classified the complications as surgical and non-surgical groups, and a significant difference was observed in both groups when comparing LHP with APR [12]. This was consistent with the results of our study. Most minor complications with significant differences between the two groups were surgical-related, including abdominal wound infection and bowel obstruction. Fortunately, all these complications were well managed, and no surgical reintervention was needed. A high incidence of small bowel obstruction (SBO) was revealed in the LHP group. The reason has not been well revealed for SBO after RC resection [23]. Generally, pelvic infection would delay the recovery of bowel function. Thus, this might be related to our study’s pelvic infection. Besides, about 10% of patients in LHP developed abdominal wound infection.

Previous studies demonstrated a higher incidence of reoperation and readmission within 30 days after LHP [18]. However, our study did not observer this, even when the statistical period was prolonged to 90 days after surgery. In our cohort, the threat of anastomotic leakage was avoided, and severe pelvic abscesses and bowel obstruction were the main events that needed further intervention. Similarly, Sverrisson’s data demonstrated a comparable rate of reoperation and readmission between LHP and APR groups [12]. Besides, we thought more attention should be paid to stoma-related complications, especially parastomal hernia, for which reoperation might be need. Thus, stoma formation is the last and also one important step in both LHP and APR. Although a higher incidence of parastomal hernia was observed in the APR group, that was not significantly different. Therefore, more clinical data are still warranted in the future for further exploration.

The multivariate analysis indicated increased blood loss as the independent risk factor for postoperative complications. This was consistent with the results in some previous studies [24, 25]. Generally, if the tumor did not invade adjacent organs or vessels, heavy blood loss was rare in both LHP and APR [26, 27]. Besides, most surgeries were performed with a laparoscopic approach, and it could further help reduce the blood loss [28]. The median blood loss was 100 ml in both groups, significantly less than in some previous studies [11]. Besides, neoCRT was less frequently performed in our cohort, because we mainly made strategies referring to Japanese guideline for a long time. However, previous studies demonstrated that neoCRT did not influence the incidence of complications between the two groups [12]. Prolonged operative time was indicated as one protective factor for bowel obstruction. This was difficult to explained because it did not conform to the common sense. We thought this mainly due to LHP was associated with shorter operative duration.

Nowadays, LHP is frequently performed for frail patients, especially those with older age or with severe comorbidity [10]. Meanwhile, APR is mainly applied to those with ultra-low location and/or sphincter involvement. In one previous survey, over 80% of surgeons preferred APR as the non-restorative procedure [5]. In our study, some patients with middle tumor height may receive anterior resection in the current situation. We thought the selection of surgical approach could be affected by several factors, including surgeons experience and preference. Besides, the difference in tumor height between the two groups should be highlighted, though this bias was also observed in previous studies. Therefore, the selection bias was the primary limitation of this study, though we have tried our best to reduce it with PSM. However, the situation still exists in which the choice should be made between LHP and APR. To our best knowledge, this was the first study to compare LHP with APR in terms of short-term outcomes with PSM. Besides, randomized clinical trials are not easy to perform regarding this issue. Thus, we thought our study still provided valuable reference to current practice.

In conclusion, our data demonstrated a comparable incidence of postoperative pelvic abscess between the two groups, and major complications. LHP is still a reliable procedure and should not be abandoned. It could serve as one alternative to APR in selected RC patients when primary anastomosis was not recommended.

## Data Availability

No datasets were generated or analysed during the current study.
